# Template-Based Recognition of Human Locomotion in IMU Sensor Data Using Dynamic Time Warping

**DOI:** 10.3390/s21082601

**Published:** 2021-04-07

**Authors:** Kim S. Sczuka, Marc Schneider, Alan K. Bourke, Sabato Mellone, Ngaire Kerse, Jorunn L. Helbostad, Clemens Becker, Jochen Klenk

**Affiliations:** 1 Department of Clinical Gerontology, Robert-Bosch-Hospital, Auerbachstr. 110, 70376 Stuttgart, Germany; Marc.Schneider@rbk.de (M.S.); Clemens.Becker@rbk.de (C.B.); Jochen.Klenk@rbk.de (J.K.); 2 Department of Neuroscience, NTNU, 7491 Trondheim, Norway; alankevinbourke@gmail.com (A.K.B.); jorunn.helbostad@ntnu.no (J.L.H.); 3 Department of Electrical, Electronic, and Information Engineering, University of Bologna, 40136 Bologna, Italy; sabato.mellone@unibo.it; 4 Department of General Practice and Primary Health Care, University of Auckland, Auckland 1023, New Zealand; n.kerse@auckland.ac.nz; 5 Institute of Epidemiology and Medical Biometry, Ulm University, Helmholtzstr. 22, 89081 Ulm, Germany; 6 Study Center Stuttgart, IB University for Health and Social Sciences, Paulinenstr. 45, 70178 Stuttgart, Germany

**Keywords:** physical activity recognition, locomotion, wearable sensors, dynamic time warping

## Abstract

Increased levels of light, moderate and vigorous physical activity (PA) are positively associated with health benefits. Therefore, sensor-based human activity recognition can identify different types and levels of PA. In this paper, we propose a two-layer locomotion recognition method using dynamic time warping applied to inertial sensor data. Based on a video-validated dataset (ADAPT), which included inertial sensor data recorded at the lower back (L5 position) during an unsupervised task-based free-living protocol, the recognition algorithm was developed, validated and tested. As a first step, we focused on the identification of locomotion activities walking, ascending and descending stairs. These activities are difficult to differentiate due to a high similarity. The results showed that walking could be recognized with a sensitivity of 88% and a specificity of 89%. Specificity for stair climbing was higher compared to walking, but sensitivity was noticeably decreased. In most cases of misclassification, stair climbing was falsely detected as walking, with only 0.2–5% not assigned to any of the chosen types of locomotion. Our results demonstrate a promising approach to recognize and differentiate human locomotion within a variety of daily activities.

## 1. Introduction

Physical activity (PA) is a core part of health and the beneficial effects of a physically active lifestyle on a variety of health parameters have been investigated in many studies [1]. Furthermore, low physical activity leads to functional decline over time [2]. The increase of physical activity is a mean to prevent disability in older adults [3]. Besides these aspects, immobility leads to an increased risk of falling due to muscle weakness and balance problems with advancing years [4]. Sufficient levels of physical activity are recommended to reduce health risks [5]. With the rapid development of eHealth devices including body-worn sensor technology, data from small wearable and unobtrusive devices are more available than ever before and can provide objective and continuous measures of physical activity [6].

Various signal processing and classification techniques have been employed to recognize different types of human activities with high accuracy, such as hidden Markov models (HMM), principle component analysis (PCA), support vector machines (SVM), linear discriminant analysis (LDA) and artificial neural networks (ANN) [7]. Although human activity recognition can be successfully achieved using wearable sensors with accuracy rates up to 94%, it is still very challenging to differentiate different types of locomotion [8]. Some activities, such as walking and climbing stairs, are more difficult to identify because of the high similarity between them. As human locomotion is periodic, in most cases a patter-based approach seems to be most suitable. Due to high variability in the timing and expression of a movement sequence within each specific locomotion event, a more robust method is needed that allows a non-linear shift in time series to hold valid for sections that are similar but out of phase. For example, single strides always look quite similar regarding the overall pattern, but duration, amplitudes and timing of gait events are not identical. Ideally, the method should not require a huge amount of data to train the algorithm. In this paper, we focus on dynamic time warping (DTW), which enables the comparison and detection of similarity between two temporal sequences that vary in speed and timing by allowing an elastic shift of the time axis [9]. The DTW algorithm compensates temporal differences between sensor signals by stretching the two vectors non-linearly in order to minimize the sum of the Euclidean distances (ED) between the corresponding points.

DTW has already been applied to different kinds of time series, such as word recognition [10,11], online handwriting recognition [12], gesture recognition [13,14] and data mining [15] and seems to be a promising approach in movement recognition. Muscillo et al. presented a real-time implementation of a DTW-based classification method to recognize upper limb movements [16]. More than 60% of the movements were correctly recognized before the motor task was finished. A 90–93% correct recognition percentage was achieved for single axis and 96.5% for triaxial accelerometer data. Al-Jawad et al. [17] developed an algorithm based on DTW for multi-dimensional time series recorded by inertial sensors located at the lower back. The algorithm was tested on 10 healthy subjects and 20 Parkinson’s patients who performed the Timed Up and Go assessment. The test showed that DTW can successfully extract time information of the transitions and turns and thereby demonstrated that DTW can be suitable in activity recognition. Renggli et al. used a template matching approach based on dynamic time warping to identify steps for gait assessments in laboratory and real-world environments [18]. Dot et al. suggested a template-based approach using DTW and a single step template of gyroscope data for step recognition in younger adults [19]. The results implied that younger, healthy subjects share a common gait pattern, so that steps of this population can be very accurately detected by an average template or piecewise-affine template. Tan et al. tested several classification methods for time series with varying length and their results showed that DTW combined with a nearest neighbor classifier demonstrated state-of-the-art performance and is considered one of the best current choices for time series classification [20].

Inertial sensor data for algorithm development and validation studies are often recorded under laboratory and supervised conditions following a structured protocol, which can lead to unnaturally performed movements. This is mainly due to: the spatial limitations of the laboratory environment; the scripted activities; and behavioral differences due to the awareness of being observed, known as the Hawthorne effect [21]. Renggli et al. demonstrated that especially older adults walk differently in laboratory conditions compared to real-world conditions [18]. Furthermore, real-world conditions increased the difference in several gait parameters between younger and older adults. Implementing a fully free-living protocol to capture totally natural behavior is difficult due to the wide variety of activities, requiring long monitoring periods to obtain adequate data for each activity of daily living. Furthermore, it is complicated to provide a gold standard (e.g., video recording), which does not affect behavior and complies with privacy requirements. In order to generate a gold standard physical activity dataset, Bourke et al. [22] harvested inertial sensor data from 20 independent living older adults who wore body-worn sensors and who were also video-recorded performing a semi-structured supervised task as well as a free-living unsupervised task-based protocol. The ADAPT dataset is a comprehensive reference dataset of representative activities from older adults that matches the target population with an increased fall risk. ADAPT provides the possibility of using recorded raw inertial sensor data together with the activity labels based on video data for the development and validation of new activity classification algorithms.

In this study we aim to develop, validate and test a new method of locomotion classification by applying DTW to detect certain types of human locomotion, such as walking and stair climbing, based on inertial sensor data recorded on the lower back of older adults in a free-living environment using the ADAPT dataset.

## 2. Materials and Methods

### 2.1. Subjects

A detailed description of the participants included in the ADAPT dataset is presented in Bourke et al. [22]. In summary, 20 older adults were recruited from a senior citizen center in Trondheim. Inclusion criteria was: over 65 years of age; able to walk 100 m without a walking aid; able to follow oral instructions; and be living independently. In our work, one dataset was excluded due to missing information, resulting in data from 19 participants being included. Furthermore, in two sets of participant data it was not possible to use the entire signal recorded during the free-living unsupervised task-based protocol due to implausible data which seemed to be caused by sensor rotations. Therefore, these sequences were excluded, however the remaining parts of the sensor signals were used.

**Figure 1:**
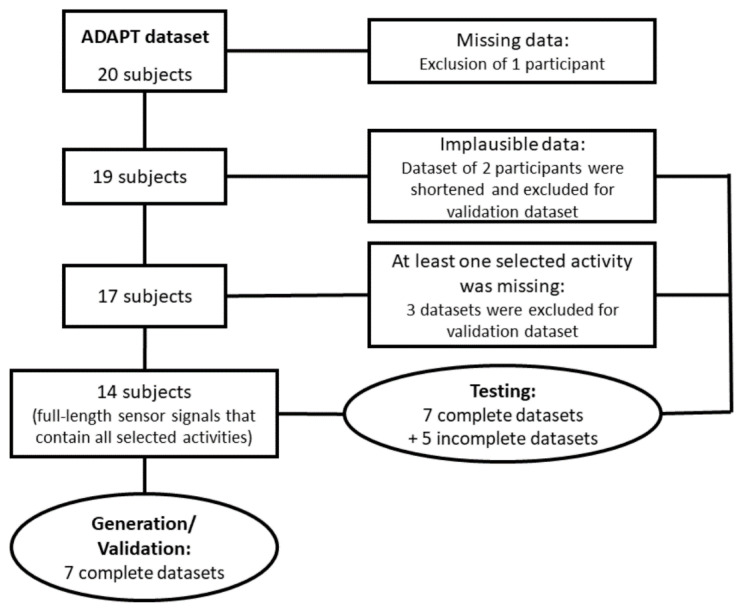
Flow chart of the data selection and allocation process.

Data of 14 participants included the activities of walking, ascending and descending stairs. Seven of them were randomly selected and used to extract a reference template database as well as for the development and validation of the activity recognition algorithm using DTW. The remaining 12 datasets were used to test the algorithm. The selection and allocation process is shown in Figure 1.

### 2.2. Sensor Set-Up

The ADAPT dataset provides a variety of different body-worn sensors and locations [22]. Within this study we used the uSense sensor at the L5 location. It consists of a tri-axial accelerometer, gyroscope and magnetometer sensors with measurement ranges of ±2 g, ±250 °/s and ±1200 µT, respectively, with a sampling frequency of 100 Hz.

### 2.3. Protocol and Performance of Activity

During the ADAPT project all subjects performed two task-based protocols: a supervised semi-structured protocol and a free-living unsupervised protocol [22]. All activities were summarized in general purpose categories: walking, shuffling, stairs (ascending/descending), standing, postural transition, sitting, lying, leaning, picking and kneeling. The semi-structured free-living protocol was recorded in the participant’s home environment using inertial sensors and a body-worn camera, GoPro Hero3+ (GoPro, Inc., San Mateo, CA, USA), worn at the chest and pointed towards the feet. Based on the video data, the performed activities were identified and the sensor data was labelled accordingly. Five raters individually labelled the data with regard to the general activity categories [23]. We focused on the locomotion activities of walking, ascending and descending stairs and the sensor signals recorded during the free-living unsupervised task-based protocol in order to develop and validate the algorithm based on daily activities performed as naturally as possible. During the unsupervised task-based free-living protocol, the subjects were asked to descend and ascend stairs, but walking up and down an inclined path was also classified as ascending or descending stairs.

### 2.4. Preprocessing Sensor Data

In this study, signals from the tri-axial accelerometer and gyroscope sensors were used. Orientation was defined as follows: z = vertical, y = mediolateral, and x = anterior-posterior. The ADAPT sensor signals were rotated so that the axes matched this format.

Furthermore, a possible misplacement of the sensor was corrected. In the case of the subject being in an upright standing position, an ideal sensor attachment at the lower back would produce values on the vertical axis that were close to 9.81$\frac{m}{s^{2}}$ whereas the perpendicular axes would show values that were close to 0 $\frac{m}{s^{2}}$. In order to correct a deviation from these values, the values during standing phases were averaged based on the “standing” labels of the ADAPT database and subtracted from the ideal value. Subsequent signals were shifted by the resulting nominal-actual value difference. Data processing and further analysis were done in MATLAB^®^ (Mathworks, 2019b).

### 2.5. Template Generation

**Figure 2:**
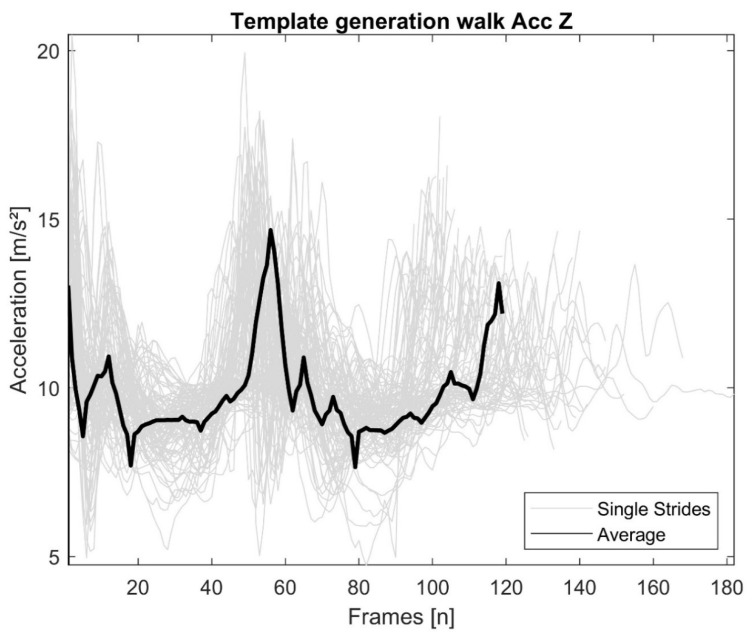
Overlay of walking stride snippets and the resulting average template calculated by the dynamic time warping (DTW) averaging method [19].

Based on the labels in the ADAPT dataset, the relevant sensor signal sections for each chosen activity (walking, ascending/descending stairs) were used. Single activity sequences were selected manually within walking and stair climbing periods, cut out based on characteristic points (single strides from the left heel strike to the subsequent left heel strike for walking and ascending/descending stairs) and stored in a database. Finally, the database provided 140 walking strides, 114 strides during ascending stairs and 97 strides during descending stairs from the seven selected subjects. Subsequently, using a DTW averaging method [24], a reference template for each chosen type of locomotion was built out of all extracted sensor signal snippets of the same activity and for each axis (Figure 2).

### 2.6. Locomotion Recognition Based on DTW

**Figure 3:**
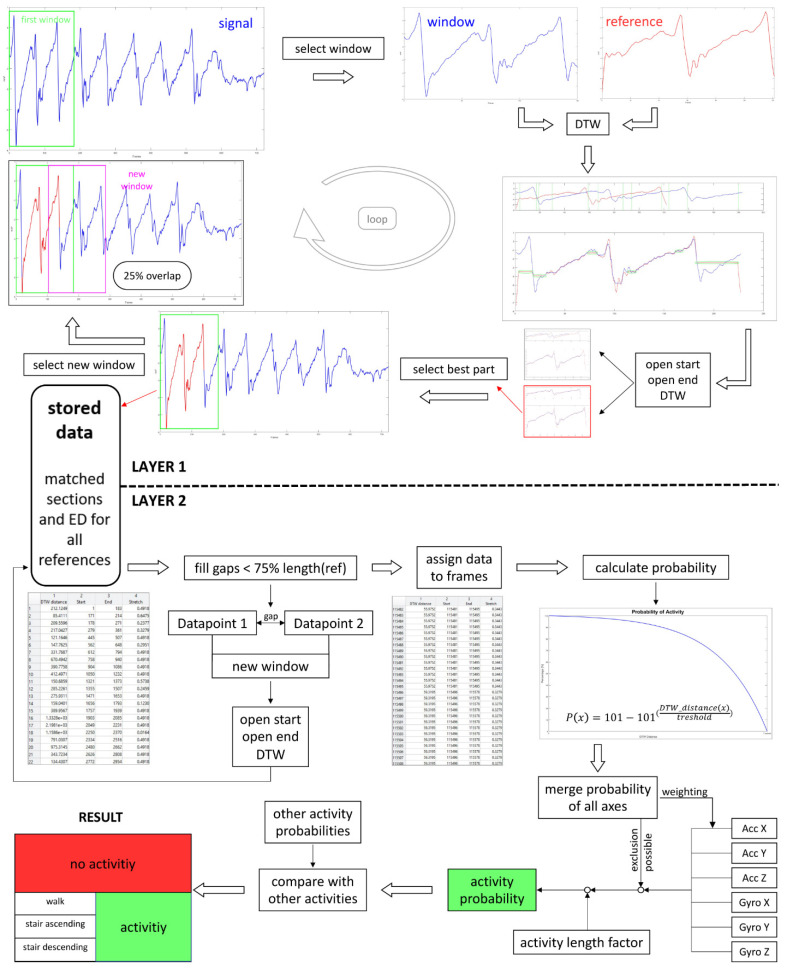
Flowchart of the locomotion recognition process.

In this approach, locomotion recognition is a two-layered process. The first layer is responsible for the comparison of the input signal with the locomotion reference templates. The similarity between the input signal and the signal snippet of the reference templates was analyzed using the built in MATLAB^®^ function *dtw.* In the second layer the resulting values for the Euclidean distance and further parameters were evaluated and the classification for most likely locomotion type was provided. Figure 3 illustrates the two-layered process. During the algorithm development and validation, values for parameters were determined based on recognition performance. The same dataset (data of seven participants) was used for the generation of the templates.

**Figure 4:**
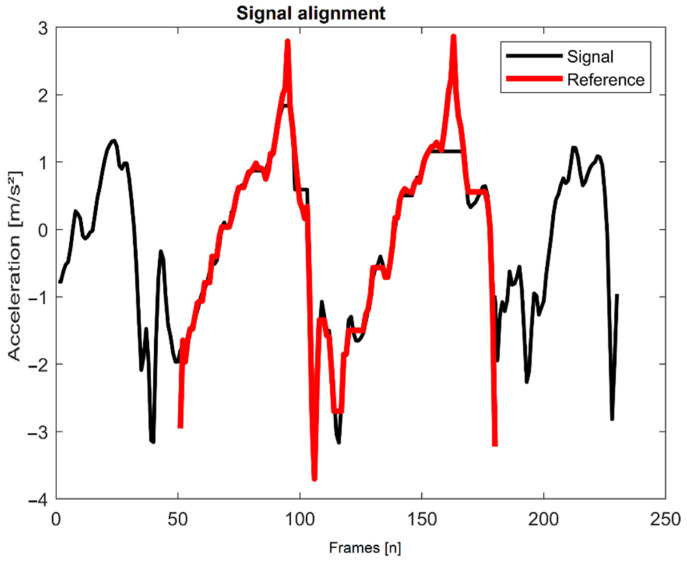
Visualization of the DTW alignment of the walking reference snippet to an input signal.

To analyze the input sensor signals, the algorithm applies a sliding window that is 1.5 times the length of the reference template. This ensured that strides of longer duration could be completely captured in a single window. Within each window the input sensor signal was compared to the locomotion reference template using DTW (Figure 4).

In order to find the section within one sliding window that matches the reference template best, stretched areas produced by DTW were identified. A high number of repetitions of the same frame were associated with a bad fit in this section. A certain number of repetitions are allowed and are often necessary to compensate for the variance in speed and timing. However, if the stretched areas were at least 10% of the reference template length, start and end points of theses sections were used as new window borders. This enabled a better framing of the single event. The prerequisite for creating a new window was that the resulting window showed at least 40% of the reference length. Shorter windows were not allowed as it turned out that shorter events were unlikely. The sensor signal snippets within these new borders were again aligned to the reference template by DTW. The section that shows the smallest ED was saved together with the corresponding ED value. The end point of the identified section was used to create the subsequent window with 25% overlap. This step was repeated until the sliding window reached the end of the signal. The whole process was completed for all axes (triaxial gyroscope and accelerometer data) and all locomotion reference templates.

Since there may be gaps between the stored sections, these were analyzed again. Gaps with a length of 25–75% of the reference length were re-examined with the described process using DTW. Gaps shorter than 25% of the reference length were filled with the values of the results of the bordering sections. The smallest value for the ED was always used. This is also the case when there was an overlap. Using this approach, an ED could be assigned to each frame.

As a next step, for each frame and each axis a probability of each locomotion type was calculated based on the ED.

(1) $$P\left(frame\right)=101-101^{\frac{DTW-Distance\left(frame\right)}{Threshold_{maxED}}}\;$$

This value was calculated using a specific threshold for the maximum acceptable ED and the actual ED of the corresponding frame. In the case of a perfect match resulting in an $DTW-Distance\left(frame\right)=0$, the probability of the examined locomotion is $P\left(frame\right)=100\%$. In Table 1, the determined thresholds for the maximum acceptable ED are displayed. Frames that showed an ED higher than the threshold resulted in $P\left(frame\right)=$ 0.

In order to calculate a probability for all axes together and for all selected activities, the probabilities of each axis were combined. For all selected activities, the vertical and anterior-posterior accelerometer signals showed the most dominant patterns that only varied minimally. These two axes were used for all selected activities and were supplemented by at least two additional axes. Usage of selected axes were determined by the recognition performance of the algorithm and are displayed in Table 2. In order to keep the evaluation duration as short as possible, as few axes as necessary were used.

Since the vertical axis alone showed good results with regards to walking, it was weighted double to increase the recognition performance. In the case that at least three axes showed $P\left(frame\right)=0\;$for the considered activity, the overall probability was set to $P\left(frame\right)=0$. Furthermore, the probability was reduced by a factor ***f*** if the detected activity showed less than 50% of the reference length. This is due to the assumption that it is unlikely that the subject performed a regular stride in half the time of the reference length.

(2) $$f=\frac{2*activity\;length}{reference\;length}$$

Finally, the overall probabilities of each frame for the different kinds of locomotion were compared and the most likely type of locomotion per frame was selected as the result. If none of the selected activities could be recognized, the result was “no selected activity.”

### 2.7. Testing

The algorithm and the defined parameters were applied to the testing dataset (data of 12 participants not included in the development and validation set). The results from the DTW algorithm were compared to the activity labels included in the ADAPT dataset and were marked as correct if the detected pattern was between the manually labelled activity borders of the gold standard dataset. Sections that were labelled in the ADAPT dataset as “undefined” and “walking with transition” were excluded because it was not possible to identify which activity took place in detail and therefore the performance of the algorithm could not be evaluated.

To evaluate the performance of the algorithm, a confusion matrix was built, and sensitivity and specificity were computed. The parameters were defined as follows:

true positives: true locomotion detections by the algorithm that matched the gold standard data

false negative: un- or misdetected kind of locomotion

true negatives: true non-detections of excluded activities by the algorithm

false positives: false detected kinds of locomotion by the algorithm

Due to the fact that locomotion segments include start and end movements which might not fit to the reference patterns, the recognition process was repeated for the same dataset, with the exception that the first and last 100 frames (1 s) of walking and stair climbing periods which were excluded. These initiation and termination sequences of locomotion activity could differ from strides performed in the middle of walking and stair climbing periods.

## 3. Results

Anthropometric data of the participants are shown in Table 3. Data of 5 male and 14 female adults, ranging in age from 68 to 90 years (76.3 ± 5.7 years), body mass from 56 to 93 kg (72.8 ± 11 kg), and height from 1.56 to 1.81 m (1.67 ± 5.7 m) were used.

Table 4 shows the sensitivity and the specificity for the three analyzed activities for the entire data and a dataset where the first and last second (1 s = 100 frames) of each walking and stair climbing period were excluded from the analysis. The highest sensitivity but lowest specificity can be seen for walking. Regarding ascending and descending stairs, the specificity was between 97% and 99%. Sensitivity was 67% for ascending stairs and was slightly lower compared to walking. Regarding descending stairs, the algorithm showed the lowest sensitivity with 29%. Average sensitivity of all selected activities could be increased by excluding the first and last second of locomotion periods.

Table 5 gives a more precise insight into the recognition performance. In total, 83.5% of all frames that were labelled as walking were identified correctly, 11.5% were wrongly recognized as ascending or descending stairs and 5% were not recognized as any chosen activity. Only 0.6% of ascending stairs could not be recognized as any kind of locomotion, but only 65.2% were true positives. Regarding descending stairs, the algorithm recognized 33.3% correctly, 3.4% were not recognized at all and 63.32% were falsely detected.

## 4. Discussion

The purpose of this study was the development of a more precise approach to detect specific types of human locomotion based on IMU data harvested from a sensor attached at the lower back using a DTW-based algorithm. Although DTW has been used successfully in various fields, until now it has rarely been used in human movement recognition. Our results showed a good recognition performance for walking and ascending stairs. Specificity for ascending and descending stairs was even higher compared to walking, however, sensitivity was lower, meaning the algorithm was very good at identifying those episodes as not descending stairs (specificity), but not very good at accurately identifying those episodes as descending stairs (sensitivity). The confusion matrix shows that most misdetections appeared for descending stairs, which is in accordance with the results of Voicu et al. [8]. They used a machine learning approach that performed very well for walking, running, sitting and standing, but showed decreased accuracy of approximately 71% for descending stairs and 75% for ascending stairs. Our analysis showed that in most cases ascending and descending stairs was confused with walking. One possible reason for this could be that the patterns of going up and down the stairs tend to vary and are therefore more complicated to detect when trying to differentiate from other types of locomotion. However, the biggest difficulty in recognizing different types of locomotion is because their patterns are almost the same and the locomotion recognition algorithm struggled to find out which one of the selected activities was actually being performed. An additional reason for this result could be that during the unsupervised task-based free-living protocol the subjects were also allowed to walk up and down an incline instead of using a staircase [22]. Therefore, in rare cases, some periods labelled as “stair climbing” could be walking up and down an incline and could be recognized correctly as “walking” by the algorithm. Even though there are misdetections in specific types of locomotion, the algorithm detection performance of locomotion in general was good.

Our findings indicate that by using at least the vertical and anterior-posterior axes of the accelerometer the algorithm was able to classify most of the activities represented by the signal correctly and avoided incorrect classification of other movements. Recognition performance could be slightly improved by adding more axes, including gyroscope data. Barth et al. showed that a DTW algorithm was able to correctly segment strides from healthy as well as altered gaits based on a template when using at least two axes of gyroscope or three axes of accelerometer data from a sensor mounted laterally on the heel of the subjects’ right and left shoes [25]. Dot et al. studied a single axis angular velocity and demonstrated a high accuracy in step recognition within gait data. They also used two inertial sensors attached to the left and right foot [19]. Within our study, the sensor was attached to the lower back and therefore the locomotion pattern within the accelerometer and gyroscope signal looked different and is less reproducible relative to a pattern recorded at the foot. Due to this, we used at least four axes, and even though the sensor location at L5 position resulted in slightly lower detection rates, the algorithm showed acceptable recognition performance. Furthermore, the sensor location provides the possibility to consider activities that include movements of the upper body that cannot be recorded by sensors attached to the feet.

### 4.1. Strengths

Even with the advances in wearable technology, there are several challenges related to the analysis of human movement based on body-worn sensors. The central issue is the lack of high-quality gold-standard datasets. A remarkable strength of this study is the development and validation based on the ADAPT dataset [22]. This gold standard dataset consists of inertial sensor raw data together with corresponding video-validated activity labels. The unsupervised tasked-based free-living protocol also provides mainly naturally performed movements. Another positive aspect is that the ADAPT dataset facilitates development of the algorithm with data from the target population of older adults.

We also want to emphasize that the dataset contains different activities and the subjects could move freely without restriction during the recording. Similar to real-world conditions, percentage of time spent walking, ascending and descending stairs was relatively low compared to all other activities (about 88%) together. Other studies showing higher rates of sensitivity and specificity used data from treadmills [26] or straight-line walking [27,28], which by design increase the probability of correct detections. No turning sequences, stair climbing or more than one gait initiation phase were included in those datasets. In contrast, in our study an arbitrary number of initiations, slowing down, turning, ascending and descending stairs, as well as bending, sitting and other activities were included. Our detection algorithm had to deal with much more challenging variations and had to differentiate between very similar signal patterns. Walking and stair climbing sensitivity increased by excluding the first and last second (100 frames) of a gait or stair climbing episode. This supports our assumption that these first and last steps show a different pattern and characteristics such as lower amplitudes and lengths compared to steps in the middle of the episode which were used for template generation.

A further advantage of the DTW algorithm is that a valid reference template can be built by using a relatively small amount of data because the general curve shape is more relevant than noise and can be aligned to similar sequences in an input signal. We demonstrated that it is possible to generate a reference template for each activity with randomly picked activity snippets and that these patterns can be used to process signals of other subjects. It is not necessary to create individual templates for each subject or train the DTW algorithm with new data. Dot et al. tested different template-based approaches in young and healthy populations and showed that using a single average step template outperformed the usage of a database of different step templates [19]. Using a single template also reduced the computation time. Our population was older and the percentage of subjects with disabilities varied between the validation and testing dataset. This difference was due to random allocation. Nevertheless, performance of the algorithm was similar when applied on a validation and testing dataset. This led to the conclusion that a template-based approach also works if the movement patterns show higher variability. However, it is possible to create individual or population-specific templates for specific analysis, which might further improve the detection performance.

### 4.2. Limitations

Although DTW shows a robustness to variations in speed and timing, it can only find similarities between the reference and the input sensor signal if the sensor’s location and orientation remains unchanged. Sensor misplacements can lead to a decreased classification performance. In terms of analyzing new sensor signals that were not recorded under observation, participants could change the sensor position or orientation and thereby effect the detection accuracy. However, Kale et al. found that ±15° misplacement in each axis can be tolerated by DTW and does not affect the performance adversely [29].

A potential issue of the proposed approach is that we developed and tested the algorithm and its parameters based only on the ADAPT dataset which included only 19 subjects. No further gold standard data to validate the algorithm with another population was available. Thus, no external validity could be demonstrated and there is a risk that the algorithm shows increased recognition rates when applied to another dataset. Renggli et al. demonstrated that real-world conditions increase the differences in certain gait parameters between young and old populations [18]. However, the aim was to develop, validate and test the algorithm. With this aim, the algorithm was developed with one part of the available dataset and tested on the other part, so it was the same cohort but different subjects. Furthermore, by using the ADAPT dataset we used an already existing dataset and we had no influence on the subjects’ instructions and the definition of the labels. As it is not clear whether the subjects only used stairs during the unsupervised task-based free-living protocol when locomotion was labelled upstairs or downstairs, the algorithm has to be further validated on the basis of another dataset.

## 5. Conclusions

DTW worked relatively well to differentiate different types of human locomotion in sensor signals of the ADAPT dataset, which included a lot of different activities of daily living. To create suitable reference templates for the detection algorithm, only the signals of seven subjects were necessary. It was possible to use these templates also for the detection of the chosen activity performed by other subjects. Compared to outcomes from previous studies that focused just on walking and stride segmentation, our results were less accurate but used a more complex approach including a high variety of activities and unsupervised real-world data. To further improve the algorithm, different templates or even adaptive templates for each activity could be used (e.g., shuffling, walking over obstacles, jumping). In the future we plan to include more reference templates for other activities, such as bending and turning, as well as variations of different activities and populations. In conclusion, DTW seems to be a promising methodology for the identification of human activities based on signals from wearable sensors.

## Figures and Tables

**Table 1:** Threshold for the maximum acceptable ED for each activity and axis.

Activity	Threshold_maxED_					
	$Acc_{X}$	$Acc_{Y}$	$Acc_{Z}$	$Gyro_{X}$	$Gyro_{Y}$	$Gyro_{Z}$
**Walking**	120	70	80	13	12	21
**Ascending stairs**	110	110	120	14	14	20
**Descending stairs**	110	90	120	14	11	25

**Table 2:** Used axes for the selected activities.

Activity	Used Axes
**Walking**	${\mathrm{Acc}}_{X}$, ${\mathrm{Acc}}_{Z}$ ${\mathrm{Gyro}}_{X}$, ${\mathrm{Gyro}}_{Y}$, ${\mathrm{Gyro}}_{Z}$
**Ascending stairs**	${\mathrm{Acc}}_{X}$, ${\mathrm{Acc}}_{Y}$, ${\mathrm{Acc}}_{Z}$ ${\mathrm{Gyro}}_{Y}$
**Descending stairs**	${\mathrm{Acc}}_{X}$, ${\mathrm{Acc}}_{Y}$, ${\mathrm{Acc}}_{Z}$ ${\mathrm{Gyro}}_{Y}$, ${\mathrm{Gyro}}_{Z}$

**Table 3:** Anthropometric data of subjects.

	Sex	Age (yrs)	Height (m)	Weight (kg)	Subjects with Disabilities/Diseases That Affect Activities (n)	Number of Fallers (n)
		mean ± SD	mean ± SD	mean ± SD		
**Reference/Validation Dataset**	Male n = 2	77.5 ± 10.61	1.75 ± 7.07	80.5 ± 0.71	1	1
	Female n = 5	73.3 ± 3.58	1.66 ± 6.7	68 ± 3.65	2	2
	Total n = 7	74.6 ± 5.59	1.69 ± 7.57	72.1 ± 7.05	3	3
**Testing Dataset**	Male n = 3	78 ± 10.58	1.76 ± 4.73	82.7 ± 9.29	2	2
	Female n = 9	77.1 ± 4.17	1.63 ± 4.56	69.5 ± 12.68	6	6
	Total n = 12	77.3 ± 5.76	1.67 ± 7.05	73.1 ± 12.95	8	8

**Table 4:** Overall sensitivity and specificity of selected types of locomotion for testing dataset and dataset with exclusion of the first and last 100 frames of each walking and stair climbing period.

Activity	Walking (Result First/Last 100 Frames Excluded)	Ascending Stairs (Result First/Last 100 Frames Excluded)	Descending Stairs (Result First/Last 100 Frames Excluded)
**Sensitivity**	79.9 (80.7)%	67.5 (72.8)%	29.2 (39.2)%
**Specificity**	88.8 (91.3)%	97.4 (97.7)%	99.1 (99.1)%

**Table 5:** Confusion matrix for testing dataset and dataset with exclusion of the first and last 100 frames of each walking and stair climbing period.

Performed Locomotion	Recognized Locomotion				Total Time of Activity (Percentage of Dataset)
	Walking (Result First/Last 100 Frames Excluded)	Ascending Stairs (Result First/Last 100 Frames Excluded)	Descending Stairs (Result First/Last 100 Frames Excluded)	No Selected Activity (Result First/Last 100 Frames Excluded)	
**Walking**	83.5 (85.1)%	7.5 (7.1)%	4.01 (5.6)%	5 (2.2)%	231 min 34 s (11.6%)
**Ascending stairs**	32.2 (31.4)%	65.2 (66.2)%	2 (2.2)%	0.6 (0.2)%	9 min 17 s (0.5%)
**Descending stairs**	52. (45.2)%	11.3 (13.5)%	33.3 (39.1)%	3.4 (2.2)%	4 min 43 s (0.2%)
**Others**					1757 min 10 s (87.7%)

## Data Availability

No new data were created or analyzed in this study. Data sharing is not applicable to this article. Data are available on request from the ADAPT research group.
